# Prevalence of and Risk Factors for Trachoma in Kano State, Nigeria

**DOI:** 10.1371/journal.pone.0040421

**Published:** 2012-07-06

**Authors:** Caleb Mpyet, Barka David Lass, Hadi B. Yahaya, Anthony W. Solomon

**Affiliations:** 1 Department of Ophthalmology, Jos University Teaching Hospital, Jos, Nigeria; 2 Department of Ophthalmology, Bingham University Teaching Hospital, Jos, Nigeria; 3 Department of Ophthalmology, Murtala Mohammed Specialist Hospital, Kano, Nigeria; 4 Department of Clinical Research, London School of Hygiene & Tropical Medicine, London, United Kingdom; Institut de Génétique et Microbiologie, France

## Abstract

**Background:**

In northern Nigeria, trachoma is an important public health problem, but there are currently few population-based data on prevalence of disease and no formal trachoma control programs.

**Methodology / Principal Findings:**

In Kano state, Nigeria, we conducted a population-based cross-sectional survey using multistage cluster random sampling, combining examination for clinical signs of trachoma and application of questionnaires assessing potential household-level risk factors. A total of 4491 people were examined in 40 clusters, of whom 1572 were aged 1–9 years, and 2407 (53.6%) were female. In 1–9 year-olds, the prevalence of trachomatous inflammation–follicular (TF) was 17.5% (95% CI: 15.7–19.5%). In a multivariate model, independent risk factors for active trachoma were the presence of flies on the face (OR 1.98, 95% CI 1.30–3.02); a dirty face (OR 2.45, 95% CI 1.85–3.25) and presence of animal dung within the compound of residence (OR 3.46, 95% CI 1.62–7.41). The prevalence of trachomatous trichiasis in persons aged ≥15years was 10.9% (95% CI: 9.7–12.2%). Trichiasis was significantly more common in adult females than in adult males.

**Conclusion/Significance:**

There is an urgent need for a trachoma control program in Kano state, with emphasis given to provision of good quality trichiasis surgery. Particular effort will need to be made to identify women with trichiasis and engage them with appropriate services while also taking steps to secure azithromycin for mass treatment and ensuring personal and environmental hygiene.

## Introduction

Trachoma, caused by *Chlamydia trachomatis*, is the most common infectious cause of blindness [1]. Infection with this bacterium may be manifest as a follicular conjunctivitis. Repeated infection over decades results in scarring of the conjunctivae, and eventually, in some individuals, the eyelids turning inwards, a condition known as trichiasis. In-turned eyelashes rub on the cornea resulting in corneal damage. This damage heals with scarring of the cornea: the cause of trachoma blindness [2]. Five clinical features of the disease have been included by the World Health Organization (WHO) in a simplified grading system. These signs include trachomatous inflammation–follicular (TF), trachomatous trichiasis (TT), and corneal opacity (CO) [3]. Presence of TF and/or TI represents “active trachoma”.

The WHO, aiming to eliminate trachoma as a public health problem by the year 2020 [4], has recommended implementation of the SAFE strategy. This involves Surgery for trichiasis, Antibiotics to clear infection, and promotion of Facial cleanliness and Environmental improvement to reduce transmission [2].

**Figure 1:**
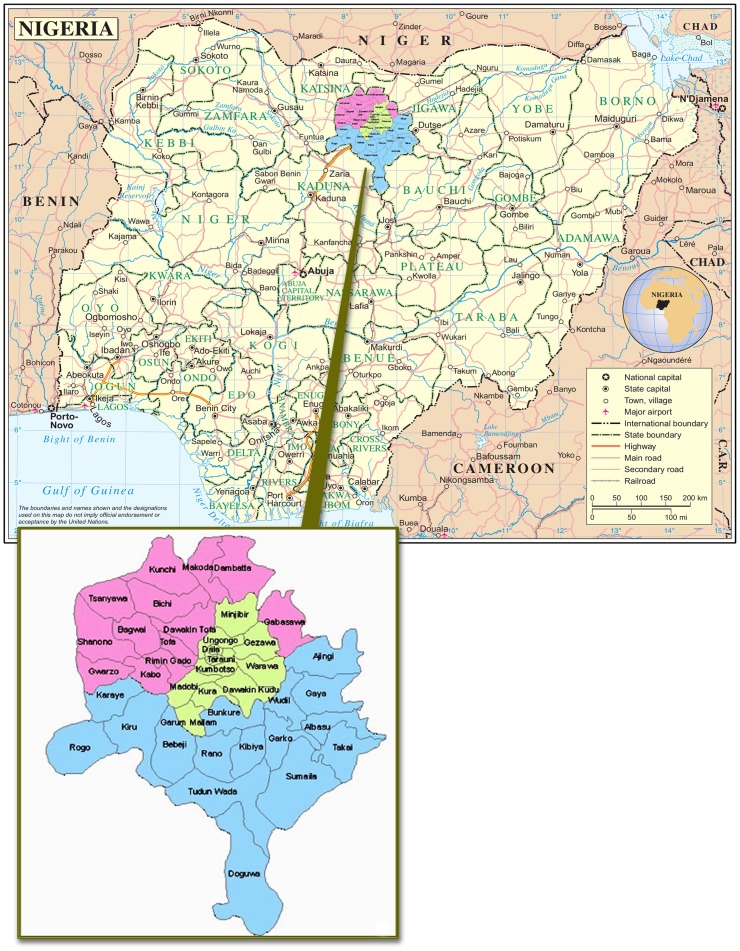
Kano State, Nigeria.

**Figure 2:**
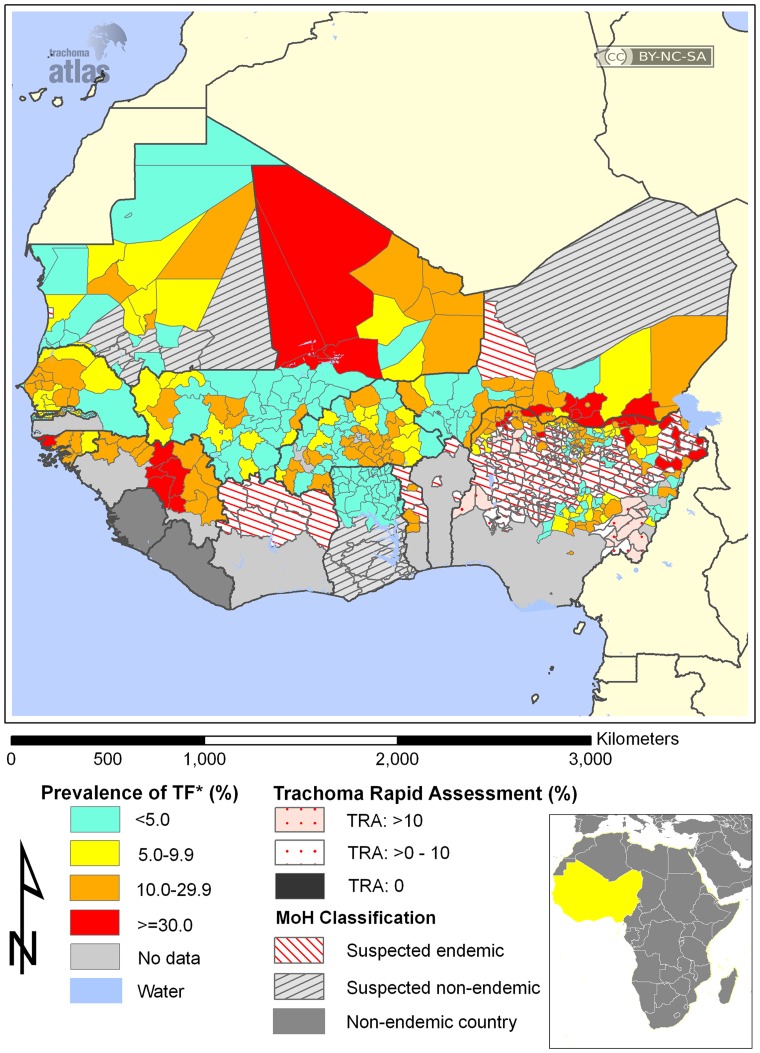
Prevalence of active trachoma in West Africa, August 2011. Adapted from The Trachoma Atlas Project (www.trachomaatlas.org) under a Creative Commons Attribution License (http://creativecommons.org).

Northern Nigeria’s Kano State (Figure 1) lies within the trachoma belt of West Africa (Figure 2). In 1995, a population-based blindness prevalence survey established trachoma as a major cause of blindness in Dambatta local government area of Kano [5]. Studies in nearby Yobe and Katsina States in Nigeria’s north have similarly found trachoma to be of public health significance [6], [7]. Despite the presence in Kano of one of the oldest eye hospitals in Nigeria, there have been no community-based efforts to assess the magnitude of the problem state-wide or to implement the SAFE strategy. We sought to estimate the magnitude of trachoma and the risk factors for disease in Kano State in order to gather evidence for implementation of control measures.

## Methods

### Ethics

The study and examination protocol were approved by the Ethics Committee of the Jos University Teaching Hospital and the Kano State Ministry of Health. Most study subjects could neither read nor write. Research assistants explained the examination protocol to each adult in a language they understood. Verbal consent for enrollment and examination was obtained from the head of each household for minors; adult gave verbal consent for their own participation. Consent was documented on individual consent forms by research assistant. Individuals with active trachoma were given two tubes of 1% tetracycline eye ointment together with instructions on its use, while adults with trichiasis were referred for free lid surgery.

### Sample Size

We followed WHO recommendations for calculating sample size in trachoma prevalence surveys [8]. Based on previous surveys in other northern states, the expected prevalence of TF in children aged 1–9 years was 25%; using a precision of 5% and a design effect of 5, the required sample size for estimating TF prevalence in children was 1440. To estimate prevalence of TT in adults aged 15 or more years we used an expected prevalence of 5%, precision of 2% and a design effect of 5, giving a required sample size of 2279 adults. For political reasons, in rural Nigerian communities it is often easier to invite the whole population (rather than population subsets) to be examined, so we decided to use an all-ages sample size of 4600 – expecting this to include about 2300 adults and 1350 children aged 1–9 years, based on the 2006 census estimates that approximately 53% of the population of Kano are aged 15 or more years, and approximately 35% are aged 0–9 years [9]. The population of the state as a whole was estimated in 2006 to be 9,012,103, composed of about 3,004,034; 0–9 year-olds, and 2,581,942 men and 2,396,894 women aged ≥15 years.

### Sampling

We used a two-stage random cluster sampling strategy to select the study population. Villages were used as clusters and 40 villages were selected from a list of all the villages in Kano state using a probability-proportional-to-size technique. In each village, a household was defined as a compound head together with all his or her dependents normally resident in the compound. Households were selected using the random walk method. A person resident in the village showed the survey team the center of the village. A pen was spun on the ground at that point and the direction the pen pointed was followed, with households in this direction being selected and eligible persons in each household examined until the required 115 persons had been examined in the village. If all households in the selected direction had been enrolled and the sample population had not yet been attained, the team returned to the center of the village to spin the pen again; this process was followed until the required numbers of persons had been examined.

### Pre-survey Training and Validation

Members of the survey team were trained in the sampling protocol, interview process, and the method for completion of survey forms; examiners were trained to grade trachoma according to the WHO simplified system [3]. For the latter, we used a set of colour slides of patients with and without trachoma, then conducted field practicals in which sets of 40 persons were examined. The findings of each examiner were compared with those of the most senior ophthalmologist (CM), with at least 80% agreement required for an examiner before he or she qualified to work on the survey. We achieved a kappa score of 0.79 for inter-observer agreement. The survey team was comprised of ophthalmologists working in trachoma endemic areas and eye nurses working in trachoma endemic communities who had previously been involved in trachoma rapid assessments.

### Data Collection

An observer assessed for facial cleanliness and the presence of flies on the face of each child examined. A clean face was defined as the absence of nasal or ocular discharge. Ocular examination was performed with the aid of penlights and ×2.5 magnifying loupes. Information on potential household-level environmental risk factors for trachoma was obtained through interview of household heads. The study was conducted in April 2008.

**Table 1:** Prevalence of signs of trachoma (WHO simplified system [3]) in children aged 1–9 years.

Sign	Frequency		Total (%)
	Females (%)	Males (%)	
TF	149 (19.7)	126 (15.4)	275 (17.5)
TI	156 (20.6)	117 (14.3)	273 (17.4)
TS	7 (0.9)	3 (0.4)	10 (0.6)
TT	0 (0)	1 (0.1)	1 (0.1)
CO	5 (0.7)	5 (0.6)	10 (0.6)

### Data Processing and Analysis

Data were entered into a Microsoft Access database, then analyzed in EPI Info version 3.3.2. Statistical tests were conducted at 5% significance level. Risk factors for active trachoma were determined by logistic regression, adjusting for clustering. Significant risk factors were assessed against each other in a multivariate logistic regression model to assess for independence.

#### Survey definitions

We used the WHO simplified grading scheme. The presence of trachomatous inflammation–follicular and/or trachomatous inflammation–intense was considered to represent active trachoma, but in accordance with international conventions, we have reported the prevalence of TF in 1–9 year-olds as our primary outcome measure for active disease.

**Table 2:** Prevalence of signs of trachoma (WHO simplified system [3]) in adults aged 15years.

Sign	Frequency		Total (%)
	Females	Males	
TF	55 (3.8)	15 (1.4)	70 (2.8)
TI	40 (2.7)	14 (1.3)	54 (2.2)
TS	59 (4.0)	42 (4.0)	101 (4.0)
TT	205 (14.0)	68 (6.5)	273 (10.9)
CO	64 (4.4)	50 (4.8)	114 (4.5)

## Results

We examined a total of 4491 people; 2407 (53.6%) were female. There were 1572 children aged 1–9 years and 2508 individuals aged 15 years or above, of whom 756 (48.1%) children and 1463 (58.3%) adults were female. The remaining 411 individuals examined were aged 10–14 years.

In 1–9 year-olds, the prevalence of TF was 17.5% (95% CI 15.7–19.5). There was only one child with TT (a 7-year-old boy); but the prevalence of corneal opacities in this age group (most of which were not related to trachoma) was relatively high at 0.6% (95% CI 0.3–1.2) (Table 1).

In subjects aged 15 years or above, the prevalence of TT was 10.9% (95% CI: 9.7–12.2) with 273 persons affected; while 114 persons (4.5%, CI: 3.8–5.5) had corneal opacities. There was a statistically significant difference in the TT prevalence between females (14.0% of those examined, 95% CI: 12.3–15.9) and males (6.5% of those examined, 95% CI: 5.1–8.2); OR 2.34 (CI 1.74–3.15), p<0.0001 (Table 2).

**Table 3:** Risk factors for active trachoma in children aged 1–9 years, determined through univariate and multivariate logistical regression.

Variable	Univariate model					Multivariate model		
	Prevalence of trachoma		OR	95% CI	p-value	OR	95%CI	p-value
Sex	Female	40.3	1.54	1.35−1.76	<0.001	0.86	0.62–1.10	0.128
	Male	29.7						
Facial cleanliness	Dirty	66.8	6.34	4.70–8.56	<0.001	2.45	1.85–3.25	<0.001
	Clean	29.1						
Animal dung in the compound	Yes	40.4	2.75	2.09–3.63	<0.001	3.46	1.62–7.41	0.001
	No	26.3						
Volume of water available for washing	Inadequate	52.5	2.18	1.73–2.73	<0.001	0.70	0.39–1.24	0.220
	Adequate	23.6						
Flies on the face	Yes	40.4	1.90	1.31–2.75	<0.001	1.98	1.30–3.02	0.002
	No	31.8						
Trash in the compound	Present	27.1	0.99	0.65–1.50	0.768	0.97	0.46–2.03	0.941
	Absent	53.3						
Toilet in the compound	Present	27.6	2.24	1.25–4.0	0.005	1.25	0.33–4.70	0.745
	Absent	40.0						

### Burden and Distribution of Disease

Based on 2006 census figures, which estimate that there are 2,581,942 males and 2,396,894 females ≥15 years State-wide [9], the calculated trichiasis backlog in Kano is estimated at 503,391 persons.

Though the sampling strategy was not constructed to provide cluster-level estimates of trachoma prevalence, there was considerable heterogeneity apparent between clusters in the proportion of individuals who had evidence of trachoma. The proportion of examined 1–9 year-old children who had TF in each cluster varied from 0% (9 clusters) to >40%. Similarly, the proportion of examined adults who had TT ranged from 0% (7 clusters) to >30%.

### Personal and Environmental Risk Factors for Disease

Independent risk factors for active trachoma (Table 3) were the presence of flies on the face (OR 1.98, 95% CI 1.30–3.02); a dirty face (OR 2.45, 95% CI 1.85–3.25) and presence of animal dung within the compound (OR 3.46, 95% CI 1.62–7.41).

## Discussion

There is a high prevalence of trachoma in Kano State. There is a pressing need to increase local capacity to identify and manage individuals with trichiasis, since such individuals are already at risk of developing trachoma-related visual impairment and blindness. Based on our data, the estimated state-wide TT surgery backlog exceeds half a million adults, and at present there are only fifteen trained TT surgeons in Kano state. There is a clear need to increase the number of eye nurses able to competently perform TT surgery and to follow-up operated cases for lid malposition or disease recurrence. Given that the burden of TT falls disproportionately on females in this setting, it will be important to ensure that case-finding strategies and service delivery models accommodate the needs of local women [10].

The state’s 17.5% prevalence of TF in 1–9 year-olds exceeds the 10% threshold for mass distribution of the antibiotic azithromycin for trachoma control [8]: plans should be made to apply for donated azithromycin for annual community distribution.

The independent risk factors for active trachoma in children in Kano State identified in this study were the presence of flies on the face, a dirty face, and the presence of animal dung in the compound of residence. The eye-seeking fly *Musca sorbens* is known to be a vector for trachoma elsewhere in sub-Saharan Africa: females of this species seek out protein-rich human ocular discharge and as they move from eye to eye, they transmit *Chlamydia trachomatis* [11], [12], [13], [14]. The finding of the presence of flies on the face at the time of examination as a risk factor for active trachoma has been documented in other settings [6], [15]; in neighboring Katsina State, this association did not quite reach statistical significance [7]. Ocular or nasal discharges (the presence of either of which we defined as constituting a “dirty face”) attract flies to children’s faces [12], and were associated in our study with the presence of active trachoma, as elsewhere [7], [15], [16]. The directionality of this association is questionable, since discharges may be *caused* by ocular chlamydial infection as well as attracting flies to introduce it. In either case, these data underline the local need for implementation of the F and E components of the SAFE strategy.

The presence of animal faeces in the compound was found to be an independent risk factor for active trachoma in this study. Animal faeces are alternative media for development of the larvae of *Musca sorbens* [13], though the flies that lay their eggs on animal waste are generally thought to be part of a different sub-population of flies to those laying eggs on human waste [13]. It may be that the custom of keeping animals close to living quarters is a marker for socio-economic status in this environment, rather than being the basis of a direct risk factor for acquiring ocular *C. trachomatis* infection.

The prevalence of trachoma has also been reported to be of public health significance in Yobe [6] and Katsina [7] States, where – as in Kano – there are no existing community-based trachoma programs. The 2005–2007 Nigeria National Blindness and Visual Impairment Survey reported that trachoma was responsible for 4.2% of all blindness in those aged ≥ 40 years nationally [17]; within Nigeria, the disease was found to be of greatest local importance as a cause of blindness in the northern “Sudan savannah” ecological zone, where it contributed 8.3% of cases of blindness in those aged ≥40 years. Although trachoma may not be a national priority for prevention of blindness in Nigeria, it still requires attention in the northern part of the country, particularly in Kano state. Implementation of the full SAFE strategy here is urgently required.
